# Associations of decreased serum total protein, albumin, and globulin with depressive severity of schizophrenia

**DOI:** 10.3389/fpsyt.2022.957671

**Published:** 2022-07-25

**Authors:** Xu Yuan Yin, Yuan Cai, Zhen Hua Zhu, Chang Ping Zhai, Jian Li, Cai Fang Ji, Peng Chen, Jing Wang, Yi Ming Wu, Raymond C. K. Chan, Qiu Fang Jia, Li Hui

**Affiliations:** ^1^Research Center of Biological Psychiatry, Suzhou Guangji Hospital, Suzhou Medical College of Soochow University, Suzhou, China; ^2^Bengbu Mental Health Center, Anhui Veterans Hospital, Anmin Hospital Affiliated to Bengbu Medical College, Bengbu, China; ^3^Changshu No. 2 People’s Hospital, The Affiliated Changshu Hospital of Xuzhou Medical University, Changshu, China; ^4^Shanghai Yangpu Mental Health Center, Shanghai, China; ^5^CAS Key Laboratory of Mental Health, Institute of Psychology, Beijing, China

**Keywords:** total protein, albumin, globulin, schizophrenia, depressive symptom

## Abstract

**Objective:**

Depression and schizophrenia (SCH) were accompanied by an acute phase response (APR) that was implicated in the alterations in total protein (TP), albumin, and globulin levels. The aims of this study are to examine serum TP, albumin, globulin levels, depressive symptoms, and their associations in patients with SCH.

**Methods:**

We recruited 34 patients with SCH and 136 healthy controls (HCs) according to the Diagnostic and Statistical Manual of Mental Disorders, Fourth Edition (DSM-IV). Psychiatric symptoms and biomarkers were assessed using the Chinese version of the Positive and Negative Syndrome Scale (PANSS) as well as the bromocresol green and biuret methods.

**Results:**

Serum TP (*F* = 46.11, *p* < 0.001, η2 = 0.19), albumin (*F* = 31.69, *p* < 0.001, η2 = 0.14), and globulin (*F* = 12.48, *p* < 0.001, η2 = 0.06) levels were lower in patients than those in HCs after adjusting for covariates. Serum TP (*r* = −0.37, *p* = 0.03) and albumin (*r* = −0.37, *p* = 0.03) levels were negatively correlated with depressive score in patients. Stepwise multivariate regression analysis showed the negative associations of depressive score with serum TP (β = −0.13, *t* = −2.92, *p* = 0.007), albumin (β = −0.23, *t* = −2.36, *p* = 0.03), and globulin (β = −0.16, *t* = −2.40, *p* = 0.02) levels in patients. Serum TP, albumin, and globulin levels exhibited the accuracies of 87.1, 70.0, and 69.4% in discriminating between patients and HCs (area under the curve [AUC]: 0.78, 0.68, and 0.77; sensitivity/specificity: 52.9%/95.6%, 55.9%/73.5%, and 76.5%/67.6%).

**Conclusion:**

Our data suggested that decreased serum TP, albumin, and globulin should be regarded as the SCH risk factors and were implicated in the depressive severity of SCH, which further provided the support for the hypothesis that SCH and depression were accompanied by the abnormal inflammatory cytokines with the APR.

## Introduction

Schizophrenia (SCH) is a highly heterogeneous disorder characterized by a series of clinical psychiatric symptoms (1). Depression has been reported to be more common in patients with SCH, and approximately 25% of patients have been found to occur in comorbid depression (2–4). Moreover, the comorbidity between SCH and depression will further result in significant social dysfunctions, life quality reduction, and suicidal risk increase (5–8). Collectively, these studies support the notion that both depression and SCH are regarded as the important targets for drug treatment as well as psychotherapy. Moreover, previous studies have reported that both depression and SCH are accompanied by an acute phase response (APR) (9–14). The APR is a systemic reaction that is implicated in the endocrine, immunologic, and metabolic systems (15–17). In addition, the APR is involved in a series of psychiatric behaviors, such as psychomotor retardation, sleep disturbance, anorexia, anergia, and lethargy (18).

Serum total protein (TP) occurs as a complex mixture of several proteins including albumin and globulin that are synthesized by the liver and blood cells (19). A previous study has explained that an APR is accompanied by the change of serum TP levels in more detail (20). Moreover, the abnormality of TP levels in the sample of different body fluids has been found to be implicated in the etiology of SCH and depression. For instance, the abnormal cerebrospinal fluid TP and γ-globulin levels have been found in 256 patients from a psychiatric unit (21). Both TP levels and albumin/globulin ratio in patients with psychiatric disorders have been reported to be significantly aberrant by detecting the sample of 278 spinal fluids (22). Serum TP, albumin, and globulin levels in patients with major depressive disorders are significantly lower than those in healthy controls (HCs), and the reductions of serum TP and albumin levels are closely implicated in the severity of depression (11). However, no studies have investigated serum TP, albumin, and globulin levels in relation to the SCH risk and depressive severity. Thus, this study aims to investigate: (1) whether serum TP, albumin, and globulin levels are closely implicated in the SCH risk; and (2) whether serum TP, albumin, and globulin levels are correlated with the SCH depressive severity.

## Materials and methods

### Subjects

The present case-control study was performed between August 2016 and December 2021. In this study, thirty-four patients with SCH were enrolled from the inpatient unit of a Suzhou City-owned Mental Hospital. The following inclusion criteria were applied: (1) patients of 18–60 years of age; (2) patients being Han Chinese; (3) two experienced psychiatrists used the Diagnostic and Statistical Manual of Mental Disorders, Fourth Edition (DSM-IV) to confirm SCH; (4) patients who have no exposure to antipsychotics in the recent 2 weeks; and (5) written informed consent and clinical assessment were obtained from each patient.

In total, 136 HCs were recruited at the same time from Suzhou local communities through the media and pamphlets advertisement. A psychiatrist used an unstructured clinical interview to assess their personal and family psychiatric histories. HCs did not have personal and family psychiatric histories.

Each subject was in good physical health, and any subject with abnormalities was excluded. Both patients with SCH and HCs did not experience drug or alcohol abuse/dependence that was examined using the lab urine tests. This study protocol and informed consent were approved by the Institutional Review Board of a Suzhou City-owned Mental Hospital, and singed informed consents were obtained from enrolled subjects.

### Clinical measurements

The detailed questionnaires as well as the samples of bloods were obtained from the subjects by two attending psychiatrists. Age, gender, education, and the age of onset were obtained from the available medical records.

Psychiatric symptoms in patients were assessed by the Chinese Version of the Positive and Negative Syndrome Scale (PANSS) (23). A previous study has reported a new PANSS five-factor model including positive symptoms (composed of four PANSS items: P1, P3, P6, and G9), negative symptoms (composed of six PANSS items: N1, N2, N3, N4, N6, and G7), cognitive symptoms (composed of three PANSS items: P2, N5, and G11), excited symptoms (composed of four PANSS items: P4, P7, G8, and G14), and depressive symptoms (composed of three PANSS items: G2, G3, and G6) (24, 25). The use of the Chinese version of PANSS was simultaneously trained for the attending psychiatrists prior to the initiation of this study.

### Total protein and albumin measurements

Blood samples without anticoagulants were collected from all subjects between 7 and 9 a.m. following overnight fasting. The serum was separated, aliquoted, and stored at −80°C in a refrigerator before laboratory assays. A HITACHI 7180 automatic biochemistry analyzer (Hitachi High-Technologies Corporation, Japan) was used to, respectively measure serum TP and albumin levels by the bromocresol green and biuret methods (commercially available kits from Medical System Biotechnology, Ningbo, China) (26, 27). Serum globulin was calculated using the formula globulin = TP – albumin (26). Serum TP and albumin levels per serum sample were measured in triplicate by the same technician who was blind to the sample’s ID and clinical information. Moreover, serum albumin in the present study was the same batch of data in the previous study of our research group (27).

### Statistical analysis

The analysis of variance (ANOVA) and chi-squared tests were used to, respectively compare the continuous and categorical variables of demographic and clinical data between patients with SCH and HCs. Serum TP, albumin, and globulin levels were compared between the two groups using ANOVA. When significant differences in serum biomarkers’ levels were observed between the two groups, further analysis would adjust for covariates. The relationships of serum TP, albumin, and globulin levels with the PANSS scores were evaluated in patients using the Pearson product moment correction coefficients, respectively. A stepwise multivariate analysis using the PANSS scores with the positive results as the dependent variables was used to investigate the impact of various variables, such as age, gender, education, the age of onset, and acute-phase protein (TP/albumin/globulin). Logistic regression analysis was further used to evaluate the classification efficiencies of distinguishing patients with SCH from HCs. The analysis of the receiver operating characteristic (ROC) curves was conducted to further plot the area under each curve (AUC), sensitivity, specificity, accuracy, and 95% confidence interval [*CI*] in discriminating between patients with SCH and HCs. All statistical analyses were performed by the R 4.1.1. The mean and standard deviation (SD) of continuous data were marked as the mean ± SD. All *p*-values (2-tailed) < 0.05 indicated the statistical significance.

## Results

### Demographic and clinical characteristics

Gender, age, and education were not different between patients and HCs (all, *p* > 0.05) (Table 1). The mean ± SD of PANSS positive symptom, PANSS negative symptom, PANSS cognitive symptom, PANSS excited symptom, PANSS depressive symptom, and the age of onset in patients with SCH were 8.82 ± 4.62, 15.71 ± 7.73, 6.71 ± 2.91, 4.91 ± 2.08, 3.88 ± 1.41, and 25.03 ± 7.14 years, respectively.

**TABLE 1:** Demographic and clinical variables in patients with schizophrenia (SCH) and healthy controls (HCs).

Variables	Patient with SCH (*n* = 34)	HCs (*n* = 136)	Statistic (*P*-value)
Age (years)	47.74 ± 10.02	44.95 ± 9.75	2.18 (0.14)
Gender (male/female)	21/13	84/52	0.00 (1.00)
Education (years)	10.88 ± 3.12	11.71 ± 3.83	1.34 (0.25)
Onset Age (years)	25.03 ± 7.14		
Positive Symptom of PANSS	8.82 ± 4.62		
Negative Symptom of PANSS	15.71 ± 7.73		
Cognitive Symptom of PANSS	6.71 ± 2.91		
Excited Symptom of PANSS	4.91 ± 2.08		
Depressive Symptom of PANSS	3.88 ± 1.41		

SCH, schizophrenia; HCs, healthy controls; PANSS, the Positive and Negative Syndrome Scale.

### Comparisons of total protein, albumin, and globulin levels between two groups

**FIGURE 1:**
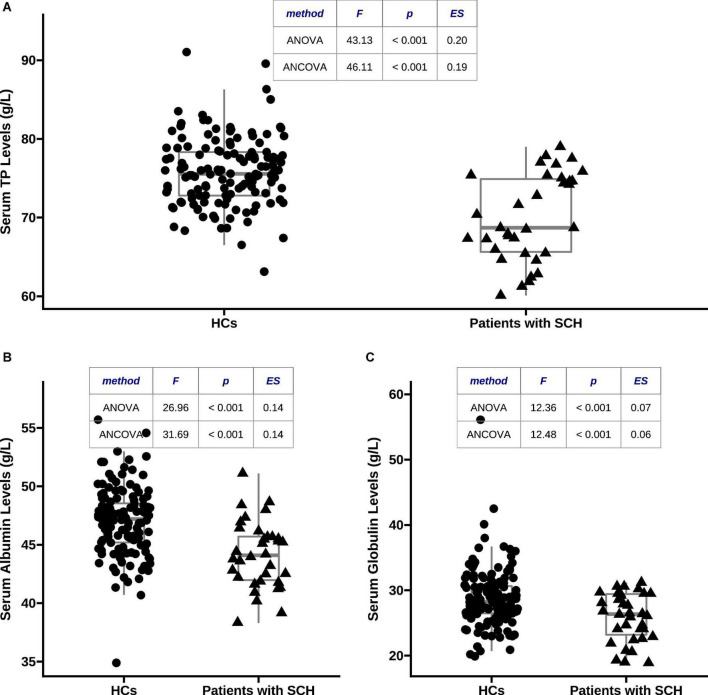
Comparisons of serum total protein (TP), albumin, and globulin levels between patients with schizophrenia (SCH) and healthy controls (HCs). **(A)** Serum TP concentrations were markedly lower in patients with SCH than that in HCs after adjusting for covariates (70.01 ± 5.52 vs. 75.84 ± 4.38, *F* = 46.11, *p* < 0.001, η2 = 0.19); **(B)** serum albumin concentrations were markedly lower in patients with SCH than that in HCs after adjusting for covariates (44.14 ± 2.87 vs. 47.01 ± 2.90, *F* = 31.69, *p* < 0.001, η2 = 0.14); and **(C)** serum globulin concentrations were markedly lower in patients with SCH than that in HCs after adjusting for covariates (25.88 ± 3.67 vs. 28.83 ± 4.54, *F* = 12.48, *p* < 0.001, η2 = 0.06). TP, total protein; HCs, healthy controls; SCH, schizophrenia; ANCOVA, analysis of covariance; ANOVA, analysis of variance.

As shown in Figure 1, serum TP (70.01 ± 5.52 vs. 75.84 ± 4.38, *F* = 43.13, *p* < 0.001, η2 = 0.20), albumin (44.14 ± 2.87 vs. 47.01 ± 2.90, *F* = 26.96, *p* < 0.001, η2 = 0.14), and globulin (25.88 ± 3.67 vs. 28.83 ± 4.54, *F* = 12.36, *p* < 0.001, η2 = 0.07) levels in patients with SCH were significantly lower than those in HCs, respectively. These differences were still significant after adjusting for covariates, respectively (TP: *F* = 46.11, *p* < 0.001, η2 = 0.19; albumin: *F* = 31.69, *p* < 0.001, η2 = 0.14; and globulin: *F* = 12.48, *p* < 0.001, η2 = 0.06).

### Associations of depressive score with total protein, albumin, and globulin levels

**FIGURE 2:**
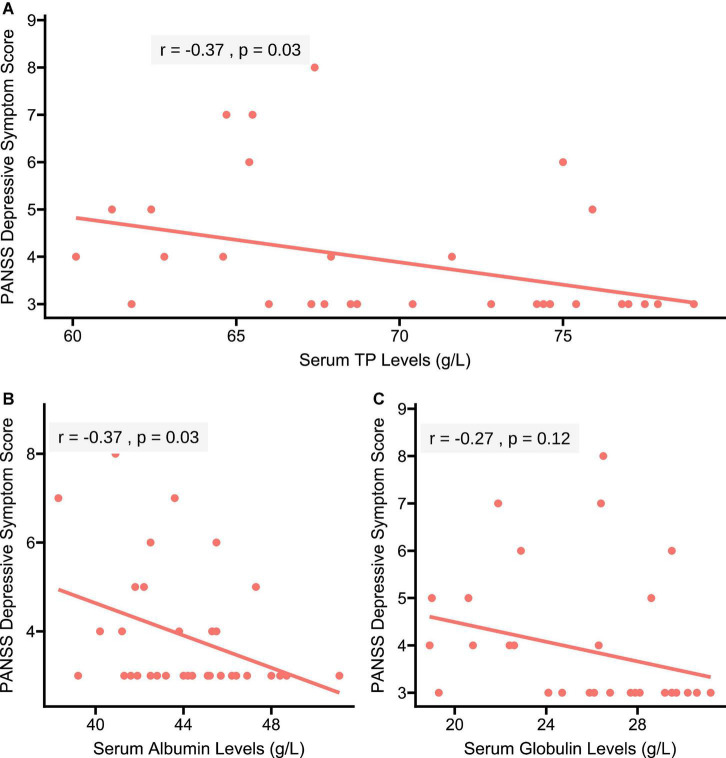
Correlations of serum TP, albumin, and globulin concentrations with depressive score in patients with SCH, respectively. **(A)** A negative correlation of serum TP concentration with depressive score was found in patients with SCH (*r* = –0.37, *p* = 0.03); **(B)** a negative correlation of serum albumin concentration with depressive symptom score was found in patients with SCH (*r* = –0.37, *p* = 0.03); and **(C)** serum globulin concentration was not correlated with depressive score in patients with SCH (*r* = –0.27, *p* = 0.12). PANSS, the Positive and Negative Syndrome Scale.

As shown in Figure 2, the Pearson correlation analysis showed the negative correlations of serum TP (*r* = −0.37, *p* = 0.03) and albumin (*r* = −0.37, *p* = 0.03) levels with depressive score in patients. However, no significant correlation between serum globulin levels and depressive score was found in patients (*r* = −0.27, *p* = 0.12).

A stepwise multivariate regression analysis further showed the significant associations of serum TP (β = −0.13, *t* = −2.92, *p* = 0.007), albumin (β = −0.23, *t* = −2.36, *p* = 0.03), and globulin (β = −0.16, *t* = −2.40, *p* = 0.02) levels with depressive score in patients with SCH, respectively (Table 2).

**TABLE 2:** A stepwise multivariate regression model of social-demographics, serum TP, albumin, and globulin determinants of the Positive and Negative Syndrome Scale (PANSS) depressive symptom score in patients with SCH, respectively.

Variables	β	t	*P*
TP (g/L)	−0.13	−2.92	**0.007**
Gender (male/female)	0.12	0.24	0.81
Age (years)	−0.03	−1.24	0.23
Education(years)	−0.03	−0.36	0.72
Age of Onset (years)	0.08	2.15	0.04
Albumin (g/L)	−0.23	−2.36	**0.03**
Gender (male/female)	−0.04	−0.07	0.94
Age (years)	−0.03	−1.14	0.26
Education (years)	−0.00	−0.04	0.97
Age of Onset (years)	0.06	1.67	0.11
Globulin (g/L)	−0.16	−2.40	**0.02**
Gender (male/female)	0.43	0.82	0.42
Age (years)	−0.02	−0.91	0.37
Education (years)	−0.06	−0.71	0.48
Age of Onset (years)	0.07	1.90	0.07

TP, total protein.

### Classifier evaluation

**FIGURE 3:**
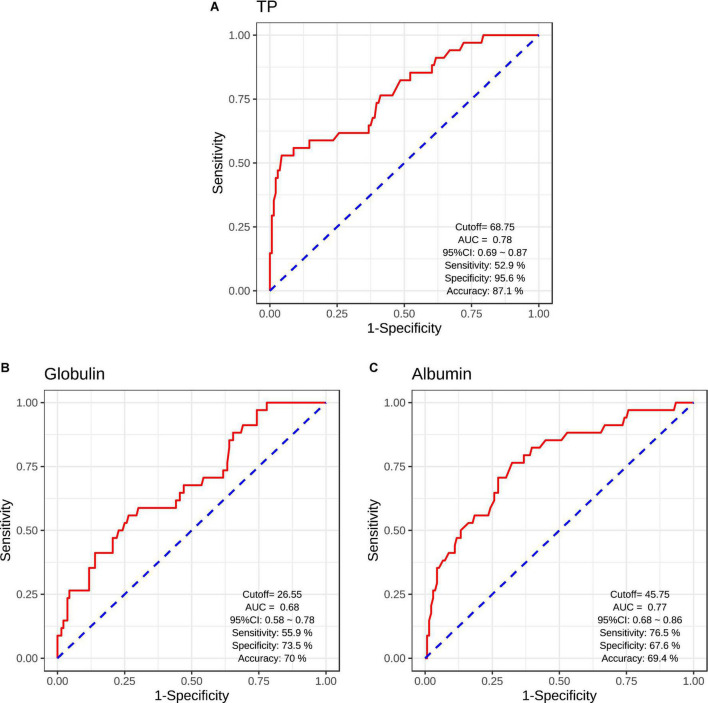
Receiver operating characteristic (ROC) curves for the classification models were identified schizophrenic diagnosis based on serum TP levels **(A)**, serum globulin levels **(B)**, and serum albumin levels **(C)**. CI, confidence interval; AUC, area under curve.

The logistic regression analysis was used to evaluate the classification efficiencies of differential diagnoses between patients with SCH and HCs. The AUCs and the accuracies of ROC curves were calculated to evaluate the classifier performance. The results of ROC analysis indicated the good performance using serum acute-phase protein (serum TP/albumin/globulin levels) (Figure 3). Serum TP levels exhibited the accuracy of 87.1% (AUC = 0.78, sensitivity/specificity = 52.9%/95.6%, cutoff = 68.75, 95% *CI*: 0.69–0.87) (Figure 3A). Moreover, serum globulin and albumin levels also exhibited the relative accuracies of 70.0% (AUC = 0.68, sensitivity/specificity = 55.9%/73.5%, cutoff = 26.55, 95% *CI*: 0.58–0.78) (Figure 3B) and 69.4% (AUC = 0.77, sensitivity/specificity = 76.5%/67.6%, cutoff = 45.75, 95% *CI*: 0.68–0.86) (Figure 3C), respectively.

## Discussion

To the best of our knowledge, this study was the first to examine the relationships of TP, albumin, and globulin with the SCH risk and depressive severity. Significant differences in serum TP, albumin, and globulin levels were found that reliably distinguished patients with SCH from HCs. Moreover, the declines in serum TP, albumin, and globulin levels were significantly associated with the depressive severity of SCH.

Previous studies have indicated that patients with SCH were closely implicated in a range of acute phase protein response (13, 14). An acute protein response might also be accompanied by the alterations in serum TP, albumin, and globulin levels (20). Collectively, these findings further indicated that serum TP, albumin, and globulin might contribute to the SCH risk. In addition, the results of the current study showed that serum TP, albumin, and globulin levels were significantly reduced in patients with SCH patients than those in HCs, which further supported that decreased serum TP, albumin, and globulin levels were involved in the risk of SCH. Several previous studies have reported that the abnormalities of TP, albumin, and globulin levels were implicated in the pathophysiology of psychiatric disorders, such as schizophrenia (21, 22). Moreover, the logistic regression and ROC analyses in our present study further confirmed that serum TP, albumin, and globulin were reliable biomarkers in discriminating between patients with SCH and HCs (Figure 3). Although serum TP, albumin, and globulin have been regarded as the important acute phase protein (11), an acute-phase protein response was still accompanied by the serious abnormalities of cytokines (e.g., interleukin-1 and interleukin-6) (28–32). In addition, the dysfunctions of immune system have been reported to be associated with the risk of SCH (33–36). For example, a meta-analysis study found that increased peripheral interleukin-1, interleukin-2, and interleukin-6 levels were implicated in the pathogenesis of SCH (37). Another meta-analysis study further indicated that interleukin-1, interleukin-6, and interleukin-12 concentrations were markedly increased in patients with the first-episode SCH in comparison with HCs (38). Taken together, significant associations of serum TP, albumin, and globulin levels with the risk of SCH might provide the support for the hypothesis that the inflammatory cytokines were related to the SCH risk.

Previous studies have found that depression may be accompanied by an APR (9–12). The abnormal levels of TP, albumin, and globulin might participate in an acute-phase protein response (20). These findings supported the notion that TP, albumin, and globulin might be implicated in the risk of depression. The present study found that serum concentrations of TP, albumin, and globulin were negatively associated with the SCH depressive symptoms, respectively. It is in accordance with the findings of previous studies reporting that there were significantly negative correlations of serum TP, albumin, and γ-globulin levels with depressive severity (11, 39). Additionally, several studies demonstrated that there were lower TP and albumin levels in subjects with depression than those in HCs, and decreased TP and albumin levels were related to depressive severity (40–42). A recent study has also found a significantly negative relationship between serum TP levels and the depressive risk in older people with function dependence in family care (43). Moreover, the decline in serum albumin levels was detected in the rat model of depression (44). The above evidence further supported that the alterations in serum TP, albumin, and globulin levels might mediate the depressive severity of SCH.

There were several limitations in the present case-control study: (1) although the sample size of subjects was relatively small, the TP (η2 = 0.19), albumin (η2 = 0.14), and globulin (η2 = 0.06) effect sizes were rather high. (2) The design of cross-sectional research. Thus, it was not clear whether there were causative associations of decreased TP, albumin, and globulin levels with the SCH depressive severity. (3) Serum globulin was calculated using the formula globulin = TP - albumin (26). Globulin has been reported to be subdivided into α_1_-, α_2_-, β-, and γ-globulin. Although serum γ-globulin levels were found to be decreased, serum α_1_- and α_2_-globulin levels were increased in patients with depressive disorder in comparison to HCs (11, 39). (4) The absence of other information, such as sleep, diet, illness duration, relapse time, body mass index, number of hospitalizations, and smoke status. For instance, a recent study has reported that serum TP and globulin levels significantly differed between SCH patients with smokers and non-smokers (26). Thus, these data should be included in the further study, which might influence the present study findings.

In conclusion, our data supported that the declines in serum TP, albumin, and globulin concentrations were significantly implicated in the SCH risk, and significant differences in serum TP, albumin, and globulin concentrations might reliably distinguished patients with SCH from HCs. Moreover, decreased serum TP, albumin, and globulin levels were involved in the depressive severity of SCH. The above findings further provided the support for the hypothesis that SCH and depression might be accompanied by abnormal inflammatory cytokines with the APR. However, these present findings are still preliminary due to the above limitations of this study. Thus, a future study is warranted to verify the present findings in an independent and large cohort of first-episode patients with naïve-drug SCH.

## Data availability statement

The datasets analyzed in this study are not publicly available. Request to access the datasets should be directed to LH, huili004100@126.com.

## Ethics statement

The studies involving human participants were reviewed and approved by the Institutional Review Board of Suzhou Guangji Hospital, Suzhou Medical College of Soochow University. The patients/participants provided their written informed consent to participate in this study.

## Author contributions

XY, YC, ZZ, and LH contributed to the overall design of this study, conducted the statistical analysis, and wrote the first draft of manuscript. XY, ZZ, JL, CJ, PC, YW, and JW recruited the subjects and performed the clinical assessment. LH generated the idea of this study and edited the manuscript. QJ and LH wrote the protocol of the study and provided funding for this study. CZ and RC commented on the manuscript critically. All authors approved the final manuscript and the submitted version.
